# Phagocytosis by Peripheral Glia: Importance for Nervous System Functions and Implications in Injury and Disease

**DOI:** 10.3389/fcell.2021.660259

**Published:** 2021-04-08

**Authors:** Lynn Nazareth, James St John, Mariyam Murtaza, Jenny Ekberg

**Affiliations:** ^1^Menzies Health Institute Queensland, Griffith University, Southport, QLD, Australia; ^2^Clem Jones Centre for Neurobiology and Stem Cell Research, Griffith University, Nathan, QLD, Australia; ^3^Griffith Institute for Drug Discovery, Griffith University, Nathan, QLD, Australia

**Keywords:** olfactory ensheathing cell, Schwann cell, cell debris, bacteria, macrophage, neuropathy

## Abstract

The central nervous system (CNS) has very limited capacity to regenerate after traumatic injury or disease. In contrast, the peripheral nervous system (PNS) has far greater capacity for regeneration. This difference can be partly attributed to variances in glial-mediated functions, such as axon guidance, structural support, secretion of growth factors and phagocytic activity. Due to their growth-promoting characteristic, transplantation of PNS glia has been trialed for neural repair. After peripheral nerve injuries, Schwann cells (SCs, the main PNS glia) phagocytose myelin debris and attract macrophages to the injury site to aid in debris clearance. One peripheral nerve, the olfactory nerve, is unique in that it continuously regenerates throughout life. The olfactory nerve glia, olfactory ensheathing cells (OECs), are the primary phagocytes within this nerve, continuously clearing axonal debris arising from the normal regeneration of the nerve and after injury. In contrast to SCs, OECs do not appear to attract macrophages. SCs and OECs also respond to and phagocytose bacteria, a function likely critical for tackling microbial invasion of the CNS via peripheral nerves. However, phagocytosis is not always effective; inflammation, aging and/or genetic factors may contribute to compromised phagocytic activity. Here, we highlight the diverse roles of SCs and OECs with the focus on their phagocytic activity under physiological and pathological conditions. We also explore why understanding the contribution of peripheral glia phagocytosis may provide us with translational strategies for achieving axonal regeneration of the injured nervous system and potentially for the treatment of certain neurological diseases.

## Introduction

Phagocytosis plays a crucial role in the development, growth and maintenance of both the CNS and the PNS. Some of the key phagocytic events include clearance of apoptotic neurons and cells undergoing necrosis or programmed cell death, as well as clearance of debris arising from pruning of axons and dendrites (Ashwell, 1990; Bishop et al., 2004; Sierra et al., 2010; Chung et al., 2013; Squarzoni et al., 2014; Amaya et al., 2015; Nazareth et al., 2015a, 2020; Mosser et al., 2017). Aging impairs efficient phagocytosis, and diminished phagocytosis has been linked to several CNS pathologies, including neurodegenerative diseases such as Alzheimer’s disease, Parkinson’s disease, multiple sclerosis, and amyotrophic lateral sclerosis (reviewed by Jung and Chung, 2018; Galloway et al., 2019; Tremblay et al., 2019; Valles et al., 2019). Impaired phagocytosis is also a problem after CNS injuries, including spinal cord injury (SCI) and traumatic brain injury (Miklossy and Van der Loos, 1991; George and Griffin, 1994; Becerra et al., 1995; Liu et al., 1995; Buss et al., 2004; Kotter et al., 2006). After peripheral nerve injury, however, removal of cellular and myelin debris is efficient and strongly aids regeneration (Stoll et al., 1989; Fernandezvalle et al., 1995; Liu et al., 1995; Perry et al., 1995; Niemi et al., 2013; Soares et al., 2014). Phagocytosis is also vital for protecting the nervous system from infection, with increased CNS infection rate or severity reported in immunocompromised patients (Nau et al., 2014).

The ability of a phagocyte to remove unwanted, dying, or aberrant cells is a complex and highly dynamic process with extremely precise recognition and degradation at the molecular level and involving multiple receptors and co-receptors. Due to the presence of the blood-brain barrier (BBB) and the glia limitans layer, the immune cell composition differs between the CNS and the PNS. In the past, the CNS was thought to be immunoprivileged, but it is now well known that this is not the case and instead the brain exhibits compartmentalized immune responses (Korin et al., 2017, 2018; Van Hove et al., 2019). With the re-discovery of the meningeal lymphatic system, we now know that these vessels serve as a direct conduit between the CNS and the peripheral immune system (Aspelund et al., 2015; Louveau et al., 2015; Absinta et al., 2017). Under normal physiological conditions, the CNS parenchyma is largely devoid of peripheral immune cells and most of the phagocytic activity is mediated by resident glia (microglia and astrocytes). Microglia are considered the macrophages of the brain and are the key CNS phagocytes. Small populations of T-cells, B-cells, NK-cells and dendritic cells also exist in the CNS, with distinct roles in the compartmentalized immune responses (Korin et al., 2017). The meninges surrounding the CNS also contain circulating immune cells which can enter the CNS after disruption of the BBB (Prinz and Priller, 2017; Papadopoulos et al., 2020).

Similar to the BBB, the peripheral nerves are surrounded by a physiological barrier known as Blood Nerve Barrier (BNB) that separates the peripheral nerve axons from the blood stream. This barrier is similar to the BBB with the exception of the astrocytes that form the glia limitans (Kuczynski, 1980; Fraher et al., 1988; Nugent et al., 1991; Nazareth et al., 2019). The outermost layer of this barrier consists of an epineurium that is mainly made up of collagen fibers, blood vessels and adipocytes (Thomas, 1963; Gamble and Eames, 1964; Ushiki and Ide, 1990; Stolinski, 1995). This encloses the perineurium consisting of collagen fibers and concentric layers of cells called perineurial cells (Shanthaveerappa and Bourne, 1963; Burkel, 1967; Akert et al., 1976). The perineurium in turn envelops the endoneurium consisting of Schwann cell-axonal structures, fibroblasts and resident macrophages (Shanthaveerappa and Bourne, 1963; Bonetti et al., 1993; Reina et al., 2015; Wolbert et al., 2020). After the breakdown of the BNB in the case of an injury or infection, peripheral immune cells (particularly macrophages) are recruited to the peripheral nerves (Perry et al., 1987; Taskinen and Roytta, 1997; Tofaris et al., 2002; Mueller et al., 2003; Beirowski et al., 2004). The peripheral nerve resident macrophages known as endoneurial macrophages, along with the glial cells contribute to the initial phagocytic clearance and aid in nerve-regeneration following injury (Mueller et al., 2003; Wang et al., 2020). However, in the unperturbed PNS, the resident glia are the main phagocytes (Bishop et al., 2004; Wu et al., 2009; Su et al., 2013; Nazareth et al., 2015a). The phagocytic activity of these glia is indispensable for development and normal physiological homeostatic maintenance. Glia are also one of the first responders to PNS injury (Chuah et al., 1995; Perry et al., 1995; Niemi et al., 2013; Su et al., 2013; Nazareth et al., 2015a). A list of phagocytic receptors and co-receptors involved in glia phagocytosis is described in Table 1.

**TABLE 1:** Phagocytic receptors and co-receptors involved in glia phagocytosis.

Receptor	Ligands	CNS phagocytosis	SC phagocytosis	OEC Phagocytosis	References
TAM receptors (Tyro3, Axl, Mertk)	PS via bridging molecules (Gas6, Protein S)	Axl: Microglial phagocytosis of apoptotic neurons and myelin. Astrocytic phagocytosis of cellular debris. Mertk: Microglial phagocytosis of apoptotic cells and cellular debris, live neurons and myelin. Astrocytic phagocytosis of apoptotic cells, cell debris and synapses	TAM receptors upregulated in SCs after PNI and involved in myelin phagocytosis	Tyro3 expression reported in olfactory bulb. Unknown if expressed by OECs or their role in phagocytosis	Axl: (Schulz et al., 1995; Weinger et al., 2011; Lutz et al., 2017; Tufail et al., 2017; Konishi et al., 2020) Mertk: (Chung et al., 2013; Neher et al., 2013; Healy et al., 2016; Lutz et al., 2017; Damisah et al., 2020) Tyro3: (Schulz et al., 1995)
Phosphatidyl serine receptor	PS	Microglial phagocytosis of apoptotic neurons	SCs recognize PS exposed on necrotic targets. Engulfment potentially via this receptor	Phagocytosis of neuronal debris and potentially necrotic cells	CNS Glia: (De Simone et al., 2002) SCs: (Nazareth et al., 2020) OECs: (Hao et al., 2017; Nazareth et al., 2020)
MFG-E8	PS; is a bridging molecule for integrin receptors α_*v*_β_3_ or α_*v*_β_5_	Microglial phagocytosis of synapses, apoptotic and live neurons. Astrocytic phagocytosis of myelin	Phagocytosis of axonal debris	Phagocytosis of apoptotic neurons	CNS Glia: (Fricker et al., 2012; Neher et al., 2013; Mills et al., 2015; Choudhury et al., 2020) SCs: (Shacham-Silverberg et al., 2018) OECs: (Li et al., 2017)
Complement receptors:	Via opsonins CR1: C1q, C3b, C4b, MBL, C3bi CR3: C3, C1q CR4: iC3b	All CRs expressed by microglia, Microglia CR1 involved in phagocytosis of Aβ(1-42); CR4 phagocytosis of apoptotic cells CR3 for synapses, myelin, apoptotic cells, infectious agents and Aβ Astrocytes express CR1; when activated (A1) also complement components (C1q, C1r, C1s, C3, and C4)	CR1is expressed on SCs during early development, pre-myelination. Potential role in myelin phagocytosis CR3 is upregulated in SCs after exposure to *E. coli* LPS. Potential role in pathogen phagocytosis. C3 production reported on SC *in vitro* challenge with *M. leprae*.	OECs express components of the complement system including C3, C1q and factor H.	CNS Glia: (Gasque and Morgan, 1996; Reichert and Rotshenker, 2003; Kaur et al., 2004; Stevens et al., 2007; Rotshenker et al., 2008; Griffiths et al., 2009; Choucair-Jaafar et al., 2011; Crehan et al., 2012, 2013; Schafer et al., 2012; Stephan et al., 2012; Weinstein et al., 2015; Choudhury et al., 2020) SCs: (Vedeler et al., 1990; Dashiell et al., 1997; Ramaglia et al., 2007) OECS: (Franssen et al., 2008)
Fcγ receptors	IgG	Expressed by microglia where they have roles in phagocytosis of opsonised cells, myelin, fibrillar Aβ and microbes, Astrocytes express FcγRIa, FcγRIIa, FcγRIIb, FcγRIIIa	FcγRI and FcγRIII expression reported in SCs. FcγRII, FcγRIII upregulation occurs post- injury and during early developmental stages (before myelin initiation).		CNS Glia: (Mosley and Cuzner, 1996; Koenigsknecht and Landreth, 2004; Lunnon et al., 2011; Fuller et al., 2014; Chauhan et al., 2017; Stamou et al., 2018) SCs: (Vedeler et al., 1990; Zhang et al., 2014; Dhiman et al., 2019)
RAGE	PS, HMGB1, Aβ, Advanced glycation end products (AGEs)	Upregulated in microglia when challenged with HMGB1 (secreted by necrotic cells), binds Aβ resulting in neurotoxicity. Astrocyte phagocytosis of Aβ	SCs upregulation of RAGE mRNA when exposed to HMGB1. Potential role in necrotic cell phagocytosis		CNS Glia: (Yan et al., 1996; Jones et al., 2013; Zhang S.T. et al., 2020) SCs: (Man et al., 2015)
MEGF-10	PS; via bridging molecule C1q	Astrocytic phagocytosis of apoptotic cells, cell debris and synapses	Upregulated in after PNI. Role in phagocytosis not known		CNS Glia: (Chung et al., 2013; Iram et al., 2016; Morizawa et al., 2017; Konishi et al., 2020) SCs:(Napoli et al., 2012; Lutz et al., 2017)
LRP-1/CD91	Myelin, Aβ	Microglial uptake of myelin, Aβ, apoptotic cells and viable neurons. Astrocyte phagocytosis of myelin, Aβ	Upregulated after injury. Potential role in myelin phagocytosis		CNS Glia: (Gaultier et al., 2009; Fricker et al., 2012; Liu et al., 2017) SCs: (Flutsch et al., 2016)
Toll-like receptors (TLRs)	Pathogenic ligands (PAMPs), endogenous ligands present on necrotic cells (DAMPs) and injured axons	TLRs 1-9 expressed by microglia. TLRs 2,4,7,9 involved in microglial phagocytosis of fibrillar Aβ42 and various TLRs in recognition of pathogens. TLR stimulation enhances microglial phagocytosis of pathogens myelin antigen presentation apoptotic cells and axonal debris. Astrocytes express TLR3 + low levels of TLR1-5/9. Upregulated in response to cytokines, bacterial ligands and infection *in vivo*.	SCs express TLR1-4, 6, and TLR7. TLR2 and 4 upregulated in after PNI and crucial for WD. TLRs upregulated in SCs after challenge with bacterial ligands and infection *in vivo*	OECs express TLR2 and 4 in response to challenge with bacterial ligands	CNS Glia: (Bowman et al., 2003; Olson and Miller, 2004; Jack et al., 2005; Farina et al., 2007; Lee and Landreth, 2010; Lee et al., 2013; Rajbhandari et al., 2014) SCs:(Oliveira et al., 2003; Boivin et al., 2007; Goethals et al., 2010; Mattos et al., 2011) OECs: (Vincent et al., 2007; Hao et al., 2017)
NOD-like receptors (NLRs)	PAMPs, DAMPs	NLRs upregulated in microglia and astrocytes on bacterial challenge resulting in inflammatory, anti-pathogenic response (Chauhan et al., 2009)	NLRs, nucleotide-binding oligomerization domain-containing protein 1 (NOD1) and NLR Family Pyrin Domain Containing 6 (NLRP6) expressed at basal levels. NLRP1 and 3 upregulated in after injury (Ydens et al., 2015)		CNS Glia: (Chauhan et al., 2009) SCs: (Ydens et al., 2015)
C-type lectin: Mannose receptor (MR)	Mannose and fructose ligands of bacteria	Expressed by and involved in phagocytosis and potentially pinocytosis (Régnier-Vigouroux, 2003) by microglia. MR involved in pathogen phagocytosis (*S. aureus*, *C. albicans*) (Marzolo et al., 1999) resulting in antigen presentation and anti-pathogenic response.	MR involved in uptake of mannosylated ligands, resulting in antigen presentation when combined with IFN-γ. Endocytosis of *S. pneumoniae* and *M. leprae* via MR is reported	OEC endocytosis of *S. pneumoniae* via MR	CNS Glia: (Marzolo et al., 1999; Régnier-Vigouroux, 2003) SCs: (Baetas-da-Cruz et al., 2009; Macedo-Ramos et al., 2014; Acosta et al., 2018) OECs: (Macedo-Ramos et al., 2011)

*PS, phosphatidylserine; PAMPS, pathogen associated molecular patterns; DAMPS, damage associated molecular patterns; Aβ, amyloid beta.*

Based on the region of the nervous system they are present in, glia are classified as CNS and PNS glia, with specific roles tailored to their anatomical location, including distinct roles in regeneration and repair. While the PNS can regenerate, unless the injury is large in particular large-gap injuries, i.e., greater than 20 mm (Sulaiman and Gordon, 2000; Lopez-Cebral et al., 2017), regeneration after CNS injury is very limited (Miklossy and Van der Loos, 1991; Kotter et al., 2006; Vargas and Barres, 2007; Lutz and Barres, 2014; Stuve and Zettl, 2014; Mietto et al., 2015). In addition to differences in intrinsic properties of PNS *vs.* CNS neurons (Fawcett and Verhaagen, 2018), the local environment is important for the differences in regeneration between the CNS and the PNS. In particular, the inflammatory environment (Fitch and Silver, 2008) and the extracellular matrix composition (Barros et al., 2011) make the CNS less growth-permissive than the PNS. The functions of peripheral glia are also considered crucial for the capacity for PNS regeneration. These functions include neurotrophic and physical support, the ability to effectively phagocytose and clear debris, as well as modulation of the inflammatory environment (reviewed by Fregnan et al., 2012; Lutz and Barres, 2014; Barton et al., 2017; Yao et al., 2018; Gilmour et al., 2020).

However, phagocytosis does not only involve clearance of dying and damaged cells, i.e., “self,” but also elimination of “non-self” targets, particularly infectious agents (Vincent et al., 2007; Mariani and Kielian, 2009; Esen and Kielian, 2012; Masaki et al., 2014). The nervous system (both the PNS and CNS) is well protected by the physical and immunological barriers of skin and mucosae, and the CNS is further protected by other barriers including the BBB and glia limitans. There are, however, microbes that are capable of crossing these barriers. The nerves that extend between the nasal cavity and the brain, the trigeminal and olfactory nerves, constitute direct paths by which microbes may reach the CNS, however, phagocytic glia of these nerves are thought to eliminate most infectious agents. Some bacteria, viruses and parasites, however, have been shown to enter and infect the CNS via these nerves (reviewed by Dando et al., 2014; Forrester et al., 2018) and CNS invasion by certain infectious agents has been linked to the development of neurodegenerative diseases (reviewed by Itzhaki et al., 2004; De Chiara et al., 2012; Balin et al., 2018; Dehhaghi et al., 2018). Understanding phagocytosis of pathogens by peripheral glia may enable us to explore ways to protect the nervous system from infection via peripheral cranial nerves.

Here, we provide an overview of the established and emerging knowledge of the phagocytic roles of two key peripheral glial cell types, Schwann cells (SCs) and olfactory ensheathing cells (OECs). We focus on these glia as they are being trialed for cell transplantation therapies due to their regenerative potential and because they are important for protecting the CNS against infection via peripheral nerves. We have previously compared various functions of OECs and SCs that aid neve-regeneration including secretion of growth factors, immunomodulatory properties, and phagocytic ability (Barton et al., 2017; Wright et al., 2018). However, there is a lack of in-depth studies focusing on the phagocytic function of these cells particularly the molecular and cellular mechanisms involved in this intricate process. Hence, we provide a comprehensive discussion about SCs and OEC phagocytosis during development, normal physiological conditions and in pathologies including infection. We also briefly introduce the origin and physiological roles of these glia. Finally, we discuss the implications of modulating the phagocytic behavior of SCs and OECs for the development of therapies.

### Glia of the Peripheral Nervous System

The glia of the PNS originate from the neural crest and support peripheral nerves by providing physical and trophic support, myelination and maintenance of homeostasis, both under physiological conditions and after injury (Ledouarin and Smith, 1988; Ledouarin et al., 1991; Buchstaller et al., 2004; Barraud et al., 2010; Suzuki and Osumi, 2015; Perera et al., 2020). The key PNS glia are (1) SCs that populate most peripheral nerves, (2) OECs, which are present in the primary olfactory nervous system (olfactory nerve and outer layer of the olfactory bulb), (3) satellite cells in peripheral ganglia, (4) enteric glia in the intestinal tract.

Schwann cells are the most abundant and well-studied glia of the PNS. SCs and neurons are intimately connected, with symbiotic dependence on each other for growth, maturation and survival. While SCs require signals from axons to undergo differentiation, axons in turn require SCs for trophic and metabolic support, as well as for normal conduction velocity (Bhattacharyya et al., 1994; Jessen et al., 1994; Dong et al., 1995; Gavrilovic et al., 1995; Grinspan et al., 1996; Woodhoo et al., 2004; Voas et al., 2009; Viader et al., 2011; Beirowski et al., 2014; Xiao et al., 2015). SCs also have active roles in aiding repair after injury (Stoll et al., 1989; Fernandezvalle et al., 1995; Perry et al., 1995; Hirata and Kawabuchi, 2002; Arthur-Farraj et al., 2012; Napoli et al., 2012; Weiss et al., 2016).

Olfactory ensheathing cells exhibit many similar characteristics to SCs, but also have distinct roles due to the constant regeneration of the olfactory nerve (Graziadei and Graziadei, 1979; Doucette, 1989, 1990; Mackaysim and Kittel, 1991). Both SCs and OECs respond to injury by phagocytosing and clearing debris, which is important for repair (Su et al., 2013; Nazareth et al., 2015a; Lutz et al., 2017). While SCs participate in the initial clearance of cellular and myelin debris, they also recruit professional phagocytes, including macrophages and neutrophils, by upregulating secretion of a range of inflammatory molecules. The recruited cells then conduct the majority of the phagocytosis to clear the injury site (Fregnan et al., 2012; Hartlehnert et al., 2017; Lindborg et al., 2017). However peripheral nerve injuries in model organisms like *Drosophila* and zebrafish reveal that macrophage recruitment to the injury site may be independent of SCs and that macrophages might arrive to the site of injury earlier than previously thought (Rosenberg et al., 2012; Soares et al., 2014). Live-cell imaging after *in vivo* injuries in zebrafish has demonstrated that cells like perineurial glia also participate in the initial debris clean up (Lewis and Kucenas, 2014). After olfactory nerve injury, however, OECs appear to be the main phagocytes and do not recruit macrophages (at least not in large numbers) (Chuah et al., 1995; Su et al., 2013; Nazareth et al., 2015a); in fact, OECs repel macrophages in co-culture (Wright et al., 2020).

Due to their capacity to remove cell debris and to promote neuronal regeneration, both SCs and OECs have been explored as candidates to treat CNS injuries, in particular spinal cord injury, via cell transplantation therapies, with variable outcomes (reviewed by Kocsis and Waxman, 2007; Kanno et al., 2015; Yao et al., 2018; Reshamwala et al., 2019). Transplantation of these glia has also been trialed for improving the rate of PNS regeneration which is ∼1 mm/day (Sulaiman and Gordon, 2000) and for repairing large peripheral nerve injuries (Chen et al., 2006; Radtke et al., 2011; Rodrigues et al., 2012; Xia et al., 2019). In addition to developmental origin, SCs and OECs share many morphological and functional properties. However, they also display distinct differences in their interactions with axons and astrocytes, migratory properties, transcriptomic profile and innate immune functions (Lakatos et al., 2000; Li et al., 2005; Vincent et al., 2005, 2007; Nazareth et al., 2020; Perera et al., 2020). These differences between the two cell types are likely to also influence their therapeutic potential. A better understanding of the functional differences between SCs and OECs will help in the design of better therapeutic strategies that can enhance the neural repair-favorable properties of these glia. One function in particular need of further examination is the phagocytic activity of the cells (Barton et al., 2017; Wright et al., 2018).

### SCs: Origin and Physiology

**FIGURE 1:**
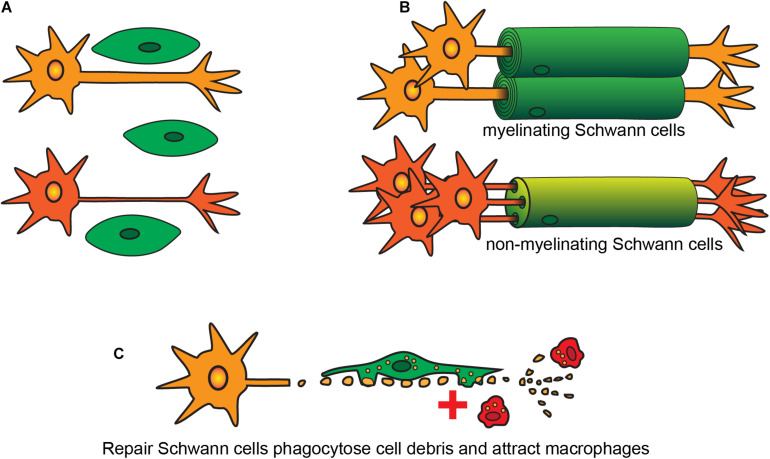
Developmental stages and types of SCs. **(A)** Immature SCs originate during embryogenesis and interact with different sized axons. **(B)** SCs become either myelinating or non-myelinating type depending on the size of the axons. **(C)** After injury both myelinating and non-myelinating SCs revert into a repair phenotype to phagocytose cell debris and to recruit macrophages.

After originating in the neural crest, SCs migrate out with the developing nerves from the neural trunk (Rickmann et al., 1985; Loring and Erickson, 1987; Ledouarin and Smith, 1988). These cells go through three key stages of development: (1) SC precursors, present during early embryonic development [embryonic day (E) 14-15 in rats, E13-14 in mice]; (2) immature SCs, existing during late embryonic development (E15-17 in rats, E13-15 in mice) (Jessen et al., 1994; Dong et al., 1995, 1999; Gavrilovic et al., 1995; Woodhoo et al., 2004); and (3) mature SCs that are present postnatally, of which there are several subtypes [the key subtypes being (i) myelinating SCs, (ii) non-myelinating SCs, and (iii) repair SCs (Figure 1)].

In both developing and adult peripheral nerves, myelination and continuous maintenance of myelin architecture around axons is indispensable for conduction of nerve impulses. On interaction with axons, immature SCs undergo numerous phenotypic changes and exit the cell cycle to form myelinating or non-myelinating SCs. Whether SCs that contact axons become myelinating or non-myelinating (the latter also known as Remak cells), depends on the axons they contact (Webster et al., 1973). The process of contacting axons is known as radial sorting, where immature SCs extend their lamellae and contact a bundle of unmyelinated axons. For axons of diameter >1 μm, the SCs establish a 1:1 contact ratio and begin myelination of these large diameter axons (Webster, 1971; Webster et al., 1973; Figure 1), with the myelin consisting of many layers of SC membranes which insulate the axons and increase conduction speed dramatically. Small-diameter axons, such as those of nociceptive neurons, remain unmyelinated and SCs contacting these axons instead become non-myelinating. In some circumstances, the SCs ensheathe multiple small-diameter axons and form an unmyelinated structure called a Remak bundle (Webster et al., 1973). However, in mammals, most non-myelinated axons undergo radial sorting with non-myelinating SCs maintaining a 1:1 contact with the small-caliber axons (Berthold et al., 2005; Sharghi-Namini et al., 2006).

Perisynaptic SCs are non-myelinating SCs present in the distal end of motor nerve terminals in the neuromuscular junctions. Their key roles are similar to those of astrocytes in the CNS, that is, modulation of synaptic activity (Robitaille, 1998; Pinard et al., 2003). After injury, both myelinating and non-myelinating SCs revert into a repair phenotype, which is discussed in the next section.

### SC Phagocytosis in Normal Physiological Conditions

Whilst the roles of SCs in neuronal support and myelination have been well studied, their phagocytic roles during development and adulthood are largely unknown. During development, more neurons are born than required, and the neurons also produce more branches than needed for effective connectivity. Excess neurons subsequently undergo apoptosis, which requires rapid clearance to prevent inflammation and antigen presentation (Dekkers et al., 2013). Whilst the contribution of SC-mediated phagocytosis to clearance of these apoptotic bodies is unknown, other peripheral glia phagocytose apoptotic neurons, including satellite glia in the developing peripheral ganglia (Wu et al., 2009) and OECs in the developing olfactory nerve (Nazareth et al., 2015a). Sequencing studies show that SCs possess several phagocytic receptors, including Tyro3-, Mertk-, and Axl- receptors (TAM receptors) and multiple EGF-like-domains-10 (MEGF10) that are upregulated after injury (Napoli et al., 2012; Weiss et al., 2016; Lutz et al., 2017). In the CNS of *Drosophila*, glia utilize the phagocytic receptor Draper (mammalian orthologue MEGF10), to engulf apoptotic neurons along with pruning of axonal and dendritic debris through various stages of development (Freeman et al., 2003; Awasaki et al., 2006). In rodents, astrocytes and microglia prune synapses and phagocytose apoptotic cells using TAM and MEGF10 receptors, both during development and in adult brains (Chung et al., 2013; Fourgeaud et al., 2016; Iram et al., 2016).Thus, it is likely that SCs can also clear apoptotic corpses and synapses and are not only phagocytic after injury but during normal physiological conditions, in particular during certain developmental stages.

Studies in *Drosophila* have also provided an insight particularly into somatoseonsory axons in contact with the skin. While glia are the main phagocytes responsible for debris and apoptotic neuron clearance in the developing *Drosophila* (Freeman et al., 2003; Awasaki and Ito, 2004; Watts et al., 2004), debris generated during dendrite pruning of sensory axons, both during development and after an injury, is phagocytosed by epidermal cells and not peripheral glia or macrophages (Han et al., 2014). Similar findings were also reported in zebrafish where epidermal cells were the primary phagocytes that cleared not only somatosensory axon debris after an injury but also peripheral axons that were misdirected to the skin (Rasmussen et al., 2015). Ablating both macrophages and peripheral glia did not affect debris clearance in this model (Rasmussen et al., 2015). In comparison, studies in the developing mammalian PNS particularly concerning phagocytic clearance are lacking. Identification of the key phagocytes may provide a better understanding of cells involved in maintaining homeostasis in the PNS.

In neuromuscular junctions, perisynaptic SCs are involved in pruning of synapses and remodeling during normal development in mammals (Bishop et al., 2004; Zuo and Bishop, 2008; Monk et al., 2015). The many axons forming connections with muscle cells during prenatal development later retract and remodel their processes (Herrera and Zeng, 2003; Wyatt and Balice-Gordon, 2003) with the retracting axons shedding structures termed axosomes, which contain synaptic organelles (Bishop et al., 2004). Perisynaptic SCs engulf the axosomes, and lysosome-bound axosomes are typically observed within perisynaptic SCs during early postnatal development (Bishop et al., 2004; Song et al., 2008). In addition to engulfing axosomes, perisynaptic SCs (and not macrophages) constitute the main cells responsible for axon pruning at the neuromuscular junction (Bishop et al., 2004; Song et al., 2008; Zuo and Bishop, 2008). Like mammals, synapse remodeling in the neuromuscular junction occurs as a part of *Drosophila* development. During this process there is generation of “waste” including presynaptic membrane shedding that generates debris and undifferentiated synaptic boutons that fail to mature (Schuster et al., 1996; Ataman et al., 2008; Fuentes-Medel et al., 2009). Unlike mammals, along with peripheral glial present in the neuromuscular junction (Awasaki and Ito, 2004) muscle cells also participate in phagocytosis of debris. Both glia and muscle cells utilize the *Drosophila* phagocytic receptor, Draper and dCed-6 (mammalian orthologue engulfment adaptor PTB Domain containing-1 (GULP); an adaptor protein) (Fuentes-Medel et al., 2009). These pathways have been previously implicated in the glial cell phagocytosis of apoptotic neurons and debris during CNS development both in insects and mammals (Awasaki and Ito, 2004; Awasaki et al., 2006; Chung et al., 2013; Iram et al., 2016). Interestingly each cell type had a specific target preference, with glia primarily responsible for presynaptic debris engulfment and muscle cells that of synaptic boutons (Fuentes-Medel et al., 2009).

After injury, SCs display both phagocytic and autophagic (“engulfment of self”) behaviors, which are covered in the next section. Whether SCs use both phagocytosis and autophagy to maintain myelin homeostasis during normal development and adulthood is unknown, however, at least autophagy appears to be involved. SCs have been shown to regulate the thickness of myelin sheaths during maturation of peripheral nerves both pre- and postnatally using autophagy (Jang et al., 2015). Transgenic mice, in which the gene encoding a protein required for autophagosome formation (autophagy related protein 7; ATG7), has been selectively knocked out in SCs, display hypermyelination in sciatic nerves resulting in abnormal peripheral nerve function postnatally (Gomez-Sanchez et al., 2015). Thus, SC phagocytosis is crucial during pre- and postnatal development, and potentially also for maintaining certain homeostatic functions in adult life.

### SC Phagocytosis in Pathological Conditions and Aging

#### Peripheral Nerve Injury

Most studies of SC phagocytosis to date have focused on responses to peripheral nerve injury, where they are important for the rapid clearance of cellular and myelin debris (Stoll et al., 1989; Perry et al., 1995; Hirata and Kawabuchi, 2002; Niemi et al., 2013; Gomez-Sanchez et al., 2015; Jang et al., 2016; Lutz et al., 2017). This occurs in a step-by-step process that has been well described, though the intricate mechanisms are still being defined. After peripheral nerve injury, the nerve distal to the site of injury undergoes disintegration, termed Wallerian degeneration, as it was first studied by Augustus Waller in Volney (1851). Destruction of axons occurs due to the initial mechanical trauma and then largely by action of calcium proteases (Touma et al., 2007). The presence of degraded axons, along with the loss of axon-glia contact, causes SCs to re-enter the cell cycle and undergo rapid proliferation (Fernandezvalle et al., 1995; Parkinson et al., 2004; Kobayashi et al., 2012; Yi et al., 2019; Welleford et al., 2020). However, the mechanisms governing this proliferation is different from that observed during development (Kim et al., 2000; Atanasoski et al., 2006). It was earlier thought that after injury, SCs merely undergo de-differentiation and transform from the myelinating/non-myelinating phenotype into an immature phenotype (reviewed by Chen et al., 2007; Jessen and Mirsky, 2008, 2016; Quintes and Brinkmann, 2017). It has, however, been shown that SCs responding to injury undergo reprogramming to down-regulate genes required for myelination and axon support, and instead up-regulate genes involved in repair and regeneration (Nagarajan et al., 2002; Bosse et al., 2006; Arthur-Farraj et al., 2012, 2017; Weiss et al., 2016; Clements et al., 2017).

The transcription factor c-Jun constitutes the main regulator of this process (Arthur-Farraj et al., 2012, 2013; Mirsky et al., 2013; Weiss et al., 2016). These reprogrammed cells constitute a distinct SC subtype – the repair SC (reviewed by Jessen et al., 2015; Jessen and Mirsky, 2016, 2019; Balakrishnan et al., 2021). Repair SCs also undergo morphological changes and are seven to ten-fold longer than immature SCs. The increase in length facilitates the formation of Büngner’s bands, which are “tracks” formed by the repair SCs surrounded by newly formed endoneurial (basement membrane) tubes, along which regenerating axons can extend and reconnect (Gomez-Sanchez et al., 2017).

Importantly, repair SCs also facilitate efficient regeneration by rapidly clearing myelin- and cell debris, by both phagocytosis and autophagy (as the myelin sheets are part of the cells themselves) (Gomez-Sanchez et al., 2015; Jang et al., 2016; Weiss et al., 2016; Lutz et al., 2017). Debris clearance occurs in peripheral nerves around day two post-injury, with around 70-80% debris cleared at 8-10 days after injury (Perry et al., 1987; Stoll et al., 1989; Soares et al., 2014). The role of SCs in debris clearance has been a source of debate over the past century. In the past, some investigators believed that SCs do not help in phagocytosis at all and that infiltrating macrophages play the major role in debris clearance (Liu, 1974; Crang and Blakemore, 1986). Other investigators suggested that this clearance may solely be a SC-mediated event (Nathaniel and Pease, 1963; Satinsky et al., 1964; Fernandezvalle et al., 1995). It is now known that the initial clearance is carried out by SCs, with myelin ovoids detected inside the cells 2 days post-injury (Hirata and Kawabuchi, 2002; Jung et al., 2011). The SCs secrete inflammatory cytokines such as interleukin-6 (IL-6), monocyte chemoattractant protein-1 (MCP-1), tumor necrosis factor-α (TNF-α), and IL-1α (Fregnan et al., 2012) which results in recruitment of macrophages to the site of injury. As hematogenous macrophages enter the injury site later, it is estimated that the initial 40-50% of the debris clearance is performed by SCs along with help from the resident endoneurial macrophages (Perry et al., 1995; Mueller et al., 2003). The exact time of arrival of hematogenous macrophages is unknown. Previous work in rodents reports macrophage arrival at the lesion site almost 2-3 days after injury, with numbers peaking between day 7 and 14 (Perry et al., 1987; Stoll et al., 1989; Taskinen and Roytta, 1997; Bendszus and Stoll, 2003; Mueller et al., 2003). One study reported the presence of infiltrating macrophages as early as 36 h after mouse sciatic nerve axotomy (Beirowski et al., 2004). However, most of these studies have been in fixed samples. *In vivo* live cell imaging in zebrafish after spinal motor nerve transection showed that macrophages arrive at the lesion site within 1-2 h post-injury (Rosenberg et al., 2012). This occurred prior to axonal degeneration. Further, the authors also indicated that immune cell recruitment in this model was SC independent. Similar results were also observed in peripheral nerve injury in *Drosophila* (performed by laser ablation of peripheral nerve of fly wing) (Soares et al., 2014). Using live *in vivo* imaging up to 2 h following injury the authors reported the migration and accumulation of hemocytes (*Drosophila* equivalent of macrophages) within 30 min post-injury (Soares et al., 2014). These immune cell numbers increased from day 2 to 7 (Soares et al., 2014). Thus live-cell *in vivo* imaging may provide a greater insight into the complex cell dynamics and response that occurs after a peripheral nerve injury (Rosenberg et al., 2012).

Further the macrophages present during the early stages of nerve injury are pro-inflammatory resembling the M1 phenotype (Peluffo et al., 2015). However, around 7-14 days post-injury the presence of M2 or anti-inflammatory macrophages are detected (Stratton and Shah, 2016). IL-10 is crucial for the switch towards the M2 phenotype. While some IL-10 is produced by SCs and fibroblasts, the majority of this cytokine is produced by the macrophages themselves (George et al., 2004).

The mechanism behind SC debris clearance is not well understood. In the past, some researchers hypothesized that the SCs take up myelin debris and release it back into the extracellular space, to in turn be phagocytosed by macrophages (Odaly and Imaeda, 1967; Liu et al., 1995). Other studies, however, indicated that SCs themselves phagocytose myelin debris, which is digested within lysosomes (Hirata et al., 1999; Hirata and Kawabuchi, 2002). These phagocytic SCs have been likened to macrophages, moving via chemotaxis towards myelin debris, also displaying myelin-recognizing receptors such as galectin-3 (thought to recognize myelin galactolipids) and macrophage markers such as the A1 antigen marker and possibly cluster of differentiation-68 (CD-68) (Oulton and Mezei, 1976; Hirata and Kawabuchi, 2002).

Again, as myelin originates from SC membranes, after injury degraded myelin has been found in autophagosomes (double-membraned vesicles) inside the cells (Thumm and Simons, 2015). Thus, the role of autophagy in the degradation of myelin by SCs has been a key focus in recent research (Gomez-Sanchez et al., 2015; Suzuki et al., 2015; Thumm and Simons, 2015; Jang et al., 2016; Belgrad et al., 2020). Autophagy has been shown to occur particularly during the active demyelination process (Gomez-Sanchez et al., 2015; Suzuki et al., 2015; Jang et al., 2016). It has also been shown that microtubule associated protein light chain 3 II (LC-3II), an indicator of autophagy, is upregulated in SCs after injury, and that autophagosomes containing myelin debris fuse with lysosomes (Gomez-Sanchez et al., 2015; Jang et al., 2016). The autophagic activity was found to be regulated by c-Jun (Gomez-Sanchez et al., 2015; Jang et al., 2016) and pharmacological inhibition of autophagy resulted in substantial inhibition of myelin degradation (Gomez-Sanchez et al., 2015).

In addition to autophagy, SCs have also been revealed to utilize receptor-mediated phagocytosis for myelin debris clearance. In a crush injury model, SCs phagocytosed myelin using two of the TAM (Tyro3, Axl, and Mer) receptors, MERTK and AXL (Lutz et al., 2017); however crush injuries generate more debris than in the commonly used transection models where only autophagy has been reported (Gomez-Sanchez et al., 2015; Jang et al., 2016). Thus, the pathways that SCs use to clear debris may depend on the type and extent of injury. Along with TAM receptors, SCs in damaged nerves also upregulate multiple EGF-like domains 10 (MEGF-10) (Napoli et al., 2012) and low-density lipoprotein receptor related protein-1 (LRP-1) (Flutsch et al., 2015, 2016). Both these receptors mediate phagocytosis of myelin and debris from apoptotic bodies by CNS glia (astrocytes and microglia) after injury in mice (Loov et al., 2012; Ponath et al., 2017; Damisah et al., 2020). Similarly, glia utilize draper (mammalian orthologue MEGF-10), for phagocytosis of debris during Wallerian Denegation after injury in the *Drosophila* CNS (Hoopfer et al., 2006). Thus, these receptors may also be involved in SC-mediated phagocytosis post-injury.

Studies in vertebrates have provided further insight that SCs and macrophages may not be the only cells involved in this process of debris phagocytosis and nerve regeneration. *In vivo* time-lapse imaging of spinal motor root axon transection (performed by laser axotomy) in zebrafish showed that perineurial glia (the cells forming the perineurium or protective casing surrounding the SC-axon complex in mature peripheral nerves reviewed by Kucenas, 2015), are also actively involved in nerve repair (Lewis and Kucenas, 2014). Previous work in rodents and *in vitro* data have indicated perineurial glia to be the cells that initiate nerve bridge formation and guide SC migration towards this bridge after injury (Schröder et al., 1993; Parrinello et al., 2010). However, the zebrafish study revealed that along with nerve bridge formation, perineurial glia also played an important role in phagocytosis of debris along with SCs and macrophages (Lewis and Kucenas, 2014). The authors also noted that the glia and macrophages tackled different areas of the injured nerve. After nerve transection the sites both proximal and distal to the site of injury undergo degeneration after which the distal stump undergoes Wallerian Degeneration (Kerschensteiner et al., 2005; Lorenzana et al., 2015). Perineurial glia and SCs were involved in phagocytosis of debris generated at the stumps occurring immediately after transection. Macrophages on the other hand were involved in phagocytosis of debris in the injury gap with additional cells recruited to clear debris generated during Wallerian Degeneration (Lewis and Kucenas, 2014). Thus, a peripheral nerve injury is a complex environment. Utilizing live *in vivo* imaging system will help understand better the cellular dynamics governing debris clearance.

We can however certainly say that SCs are active phagocytes. Along with *in* vivo studies discussed above there are numerous *in vitro* studies from as early as 1945 indicating that SCs can internalize different types of targets, including latex beads, myelin, olfactory axon debris, heat-killed *Escherichia coli* bacteria and mycobacteria (Weiss and Wang, 1945; Band A.H. et al., 1986; Band H. et al., 1986; Velasquez et al., 2018). These vastly different targets, with a wide range of sizes and surface molecules, can be internalized and processed using a variety of mechanisms. Most studies to date have only focused on internalization, without investigating the capacity and efficiency for degradation and break down (or the timeline required for the different phases of phagocytosis). Regarding debris, the mechanism of uptake is to date mostly unknown, other than that the internalization of debris is actin-mediated and involves the Rho/Rac pathways (Band H. et al., 1986; Velasquez et al., 2018). Omics-profiling of repair SCs has identified several phagocytic and endocytic receptors (such as MERTK and Cathepsin D), along with lysosomal and endosomal markers which likely aid in endocytosis, efferocytosis and myelinophagy, that are expressed by repair SCs after injury (Weiss et al., 2016). We have also recently shown that SCs can phagocytose necrotic bodies by recognizing phosphatidylserine displayed on the surface of the dying cells. We also showed that while these targets are rapidly engulfed, their breakdown is much slower in SCs than in macrophages (Nazareth et al., 2020).

Schwann cells also contain a range of other receptors important for recognition of damage-associated molecular patterns (DAMPs) released by necrotic cells, including Toll-Like receptors (TLRs); TLR2 TLR3, TLR4, TLR7, and receptor for advanced glycation end products (RAGE) (Goethals et al., 2010). A study of SCs challenged with high mobility group box 1 (HMGB1), a DAMP passively released by necrotic cells after peripheral nerve injury, resulted in upregulation of TLR2 and RAGE mRNA in SCs (Man et al., 2015). In addition to up-regulating chemoattractants, human SCs have been shown to participate in antigen presentation via MHC-II (Suzuki et al., 2015; Weiss et al., 2016).

Thus, SCs display a number of phagocytic receptors that are potentially important for phagocytosis of cellular and myelin debris (Weiss et al., 2016; Lutz et al., 2017). SCs are capable of efficiently internalizing and degrading various necrotic targets *in vitro* (Nazareth et al., 2020), however, this remains to be shown *in vivo*. Similarly, whether SCs show similar efficiency in the phagocytosis and degradation of apoptotic targets remains to be determined. In spite of their phagocytic ability, after peripheral nerve injuries i*n vivo* SCs enlist the help of various cells including perineurial glia and immune cells to phagocytose debris (Perry et al., 1987; Mueller et al., 2003; Beirowski et al., 2004; Rosenberg et al., 2012; Lewis and Kucenas, 2014; Lindborg et al., 2017).

#### Peripheral Neuropathies

Some peripheral neuropathies involving SCs include Charcot-Marie-Tooth disease (CMT), Guillain-Barré syndrome, diabetic neuropathy, and neuropathic pain. Peripheral neuropathies have also been reported in the aging population (reviewed by Verdu et al., 2000). Similar to Wallerian degeneration, these conditions are characterized by axonal degradation and demyelination. However, unlike Wallerian degeneration, nerve regeneration and remyelination is impaired either due to excessive degradation of myelin or abnormal myelin clearance (Martini et al., 2013). Thus, it is likely that perturbations of SC-mediated phagocytosis may in some way be involved in the onset and progression of peripheral neuropathies, but this remains to be investigated.

Charcot-Marie-Tooth diseases constitute a group of hereditary disorders and the manifestation of the disease is dependent on inherited genetic mutations (reviewed by Juneja et al., 2019). CMT is classified as demyelinating CMT or axonal CMT, with 80% of the cases belonging to the demyelinating form which is due to the inability of SCs to myelinate or maintain axonal myelin (Berger et al., 2006). As mentioned above, after peripheral nerve injury, both myelinating and non-myelinating SCs revert to a repair phenotype accompanied by upregulation of c-Jun (Arthur-Farraj et al., 2012, 2013). This also drives SC-mediated autophagic clearance of myelin (Gomez-Sanchez et al., 2015; Jang et al., 2016). In later stages of injury, c-Jun expression is down-regulated, and SC differentiation occurs, allowing myelination of newly generated axons (Mirsky et al., 2013; Jessen and Mirsky, 2016; Wagstaff et al., 2017). However, in CMT Type 1A (CMT1A), prolonged elevated levels of c-Jun (Hantke et al., 2014) and LC3B-II (which promotes autophagy) (Lee et al., 2018) have been reported in the SCs surrounding nerves that have not been injured. This dysregulation of SCs autophagy is thought to result in extended Wallerian degeneration, preventing remyelination of axons (Hutton et al., 2011; Lee et al., 2018). Macrophage-mediated phagocytosis of myelin has been shown to contribute to demyelination, particularly in Type 1 CMT mice. Studies on CMT1A, CMT1B and CMT1X mutant mice, have observed an increased number of macrophages near demyelinated nerves exhibiting a “foamy” appearance, due to internalization of myelin from nerves (Kobsar et al., 2005; Groh et al., 2012; Klein et al., 2015; Yuan et al., 2018). It is to date unknown if SCs in CMT also have an elevated phagocytic capacity and if this contributes to demyelination.

Neuropathic pain may arise due to peripheral nerve pathologies and injury. SC responses to PNS injury can play an important role in the progression of pain. Early activation of autophagy in SCs by the pharmacological agent rapamycin has been shown to induce rapid and increased clearance of myelin and axonal debris, promoting nerve regeneration, and reducing chronic pain in rodent pain models (Rangaraju and Notterpek, 2011; Marinelli et al., 2014). Perhaps stimulating SC phagocytosis would have similar therapeutic potential in treating neuropathic pain, however, care must be taken to not produce a strongly pro-inflammatory environment.

Similar to peripheral neuropathies, aging affects several morphological features of peripheral nerves, including loss of myelinated and unmyelinated fibers along with myelination abnormalities (Sakita et al., 2016). Impaired recovery after peripheral nerve injury has also been reported in the elderly population (Verdu et al., 2000). Matching these human studies, SCs in older mice express lower levels of c-Jun with a delay in de-differentiation into the repair SC phenotype after injury than in younger mice (Painter et al., 2014). SCs isolated from aged mice (24 months) also exhibit lower myelin clearance capacity than cells from younger mice, with a 35% decrease in myelin phagocytosis compared to cells isolated from younger animals (Painter et al., 2014). A study investigating peripheral nerve injury in aged rats also showed a decrease in phagocytic ability both in SCs and macrophages. The same study also showed that levels of anti-inflammatory factors such as IL-10, arginase-1, and CCL-2, which normally increase after PNS injury, were decreased in aged rats, potentially correlating with the ability for regeneration and debris clearance (Scheib and Hoke, 2016). Interestingly, one study showed that when sciatic nerves were grafted from old rats into younger rats their myelin clearance improved; conversely, grafting of young sciatic nerves into older rats resulted in accumulation of myelin debris. This indicates that the environment after peripheral nerve injury is crucial for debris clearance (Sakita et al., 2016). Hence, SCs play a crucial role in debris clearance after an injury and reduced phagocytic ability correlating with increasing age may be one of the contributing factors to peripheral nerve abnormalities in the older population.

In summary, SC autophagy and phagocytosis may be intimately related and both crucial for the clearance of myelin-, axon- and cell debris after an injury. It is likely that dysregulation of these functions in SCs contribute to various neuropathies. Determining the cellular and molecular mechanisms behind the autophagic and phagocytic functions of SCs and gaining a deeper understanding of these functions in injury repair, may lead to new effective treatments for peripheral neuropathies.

#### Peripheral Nerve Infections

Understanding peripheral glia phagocytosis of damaged “self” is vital in the context of injuries and neuropathies. However, an equally important arm of glial phagocytosis is that of “non-self.” Infections can lead to damage of peripheral nerves, resulting in neuropathies. Most of these pathologies are due to immune responses, in particular development of autoimmunity, for example production of antibodies targeting axons or myelin in addition to, or rather than, the infectious agent (reviewed by Neal and Gasque, 2016). However, a few pathogenic bacteria, viruses and protozoans can directly infect SCs, which can result in peripheral nerve damage (Neal and Gasque, 2016) or invasion of the CNS via cranial nerves in which SCs are found, in particular the trigeminal nerve (St John et al., 2016; Duarte et al., 2019; Nazareth et al., 2021). The intranasal branches of this nerve can serve as a direct path to CNS infection by several infectious agents, including *Streptococcus pneumoniae*, *Burkholderia pseudomallei*, *Chlamydia muridarum*, *Herpes simplex* virus, and *Listeria monocytogenes* (Shimeld et al., 2001; van Ginkel et al., 2003; St John et al., 2014, 2016; Wei et al., 2020; Nazareth et al., 2021). Thus, understanding SC phagocytosis of “non-self” is important and may lead to novel therapies, as mentioned earlier.

Schwann cells are efficient immune cells displaying several pathogen recognition receptors (PRRs) that they utilize to recognize invading pathogens. Some of these receptors include TLR 1-4, TLR 7, Nod-like receptors, RAGE, C-type lectin receptors, such as cluster of differentiation 209 (CD 209) and mannose receptors (reviewed by Ydens et al., 2013). SCs also display Fcγ receptors (FcγRII and FcγRIII) and components of the classical complement activation pathway, including complement receptor 3 (CR3) (Vedeler et al., 1989, 1990). As macrophages are known to utilize both FcγRs and CR3 to phagocytose infectious agents (Fitzer-Attas et al., 2000; Campagne et al., 2007), it is likely that SCs also utilize these receptors to recognize and engulf microbes. SCs have been also reported to produce a range of cytokines, including interleukins such as IL-6, IL-8, IL-10, IL-23, IFN-β1, IFN-γ, IL-1β, and TNF-α, and chemokines, such as CCL-2,CC-17, CC-19, CXC-11, CXCL-1, MCP-1, MIP-1α, MIP-1β in response to pathogens (Ramesh et al., 2013; Masaki et al., 2014; Dhiman et al., 2019; Nazareth et al., 2021). Secretion of these cyto- and chemokines occurs in parallel with activation of NF-κB and inducible nitric oxide (iNOS) production, in response to pathogenic ligands (Lee et al., 2007). SCs also display MHC molecules and can upregulate and display functionally active MHC class I and II structures when challenged by various pathogens (Samuel et al., 1987; Pereira et al., 1994). Thus, SCs can act as non-professional antigen-presenting cells.

Schwann cells can mount an effective anti-pathogenic immune response to combat infections (Neal and Gasque, 2016), but some microbes instead survive in SCs by manipulating components of the phagocytic pathway. *Mycobacterium leprae*, the obligate intracellular bacterium that causes leprosy, preferentially infects SCs in the peripheral nerves. There are various structures on *M. leprae* that bind to receptors on SCs allowing their entry. These include 21-kDa molecule (ML-LBP21) that binds to the α2 laminins on SCs, promoting attachment and entry of the bacteria (Shimoji et al., 1999). *M. leprae* bacteria also contain phenolic glycolipid 1 (PGL 1) that binds to mannose receptors on SCs, promoting endocytic uptake of the bacteria (Acosta et al., 2018). After binding to SCs, *M. leprae* modulates host tyrosine kinases, in particular phosphoinositide 3-kinase (PI3K), promoting entry into the cell (Alves et al., 2004). After entry, the bacterium resists endosome processing and maturation by recruiting host cell-derived lipid droplets to phagosomes that contain *M. leprae* bacteria. The genesis of these lipid droplets within the host cells also further increases SC production of prostaglandin E and IL-10, accompanied by decreased IL-12 and iNOS production (Mattos et al., 2011). Thus, *M. leprae* suppresses SC immune responses and persists within the cells.

*Mycobacterium leprae* can infect both myelinating and non-myelinating SCs. Whilst it shows a greater affinity for non-myelinating SCs, infection can lead to severe demyelination of peripheral nerves (Kumar and Sengupta, 2003). *M. leprae* also takes advantage of the plastic nature of SCs, and after infection re-programs adult SCs into progenitor-like cells. This is accompanied by upregulation of sex determining region Y (SRY) box-2 (Sox-2), seen in early developing neural crest SCs, and downregulation of SRY box-10 (Sox-10), required for SCs differentiation into the myelinating phenotype (Le et al., 2005; Finzsch et al., 2010; Masaki et al., 2013b) – consistent with de-differentiation of the cells. These progenitor-like cells are different from repair SCs, with the progenitor cells expressing both Sox-2 and Sox-10 whilst the repair cells only express Sox-2 (Jessen and Mirsky, 2016). The reprogrammed cells then transfer the bacterium to their neighboring fibroblasts, promoting dissemination within the nerves (Masaki et al., 2013a).

Bacteria that invade the CNS through the trigeminal nerve have been shown to infect SCs *in vitro*. When SCs were inoculated with *S. pneumoniae*, the bacteria utilized mannose receptors to the enter cells (Macedo-Ramos et al., 2014). *B. pseudomallei*, the bacterium that causes melioidosis (including neurological infection), is one of the microbes that can invade the brain after intranasal exposure, via both the trigeminal and the olfactory nerves (St John et al., 2014, 2016). We have found that *B. pseudomallei* infects a subpopulation of SCs, which then become multinucleated, most probably through the bacterial protein *Burkholderia* intracellular motility A (BimA), that manipulates the host actin cytoskeleton resulting in cell-cell fusion. However, some cells did not form multinucleated giant cells and contained degraded bacteria (Walkden et al., 2020). *Neisseria meningitidis* can also infect trigeminal SCs, leading to multinucleation via unknown mechanisms (Delbaz et al., 2020).

*Chlamydia pneumoniae* has been shown to infect the CNS and linked to late-onset dementia (Balin et al., 1998, 2018; Gerard et al., 2006). Since *C. pneumoniae*-infected mice rapidly develop olfactory bulb infection after intranasal exposure, the olfactory nerve has for some time been considered a likely path to the CNS for this bacterium (Little et al., 2004, 2014; Boelen et al., 2007). *C. muridarum*, another *Chlamydia* species that infects mice, is often used to model *C. pneumoniae* infections. We recently showed that mice inoculated intranasally with *C. muridarum* develop CNS infection via both the olfactory and the trigeminal nerves (Nazareth et al., 2021). *In vitro*, trigeminal SCs were readily infected by *C. muridarum*, but exhibited more resistance to infection than fibroblasts (Nazareth et al., 2021). *Chlamydiae* are obligate intracellular bacteria that live inside modified intracellular membrane compartments termed inclusions. The bacteria manipulate the host phagocytic pathway, including the actin cytoskeleton, to induce entry and suppress endosomal-lysosomal components and recruit recycling endosomes. Thus, *Chlamydiae* can live intracellularly and evade degradation by the phagocytic machinery (reviewed by Gitsels et al., 2019). How *Chlamydia* bacteria specifically modulate SC phagocytosis to survive intracellularly remains to be investigated.

*Trypanasoma cruzi*, an obligate intracellular protozoan and the causative agent for Chagas disease, can cause both PNS and CNS infections and have particular affinity towards infecting glia (Weinkauf and PereiraPerrin, 2009). *T. cruzi* contains trans-sialidase parasite-derived neurotrophic factor (PDNF) that is similar to neurotrophin-3 (NT-3). PDNF binds to and activates neurotrophic receptor tyrosine kinase C (TrkC) which allows *T. cruzi* entry into SCs (Weinkauf and PereiraPerrin, 2009). Phosphorylation of components of the MAPK, Erk1/2 and Akt pathways then occurs, preventing host cell death which allows the parasite to survive within the cells (Chuenkova and PereiraPerrin, 2009).

Thus, SCs can recognize, internalize, and produce an immune response to microbes. However, while SCs are more resistant to infection than many non-professional phagocytes (such as fibroblasts), they appear to be not as efficient phagocytes as macrophages (Nazareth et al., 2021). Intracellular pathogens, in particular, can manipulate SC plasticity and phagocytic pathways to survive within the cells. Understanding the modulation of SCs by pathogens and further manipulation of the intrinsic immune capacity of these cells holds great therapeutic potential to reduce the risk of PNS infections and potentially prevent CNS pathologies.

In summary, SCs display a range of phagocytic receptors that they use to recognize and internalize dying and damaged “self” as well as invading “non-self.” SCs actively participate in the initial debris clearance at a PNS injury site, however, they also recruit macrophages for complete removal of cellular and myelin debris. While studies of SC-mediated phagocytosis of myelin (particularly after injury) is well documented, the role of SC phagocytosis during development and in various peripheral neuropathies has not been investigated in great detail (especially in mammals). Finally, SCs can mount an immune response against infectious agents, but certain microbes can modulate the cells to instead cause infection.

### Olfactory Ensheathing Cells (OECs): Origin and Physiology

**FIGURE 2:**
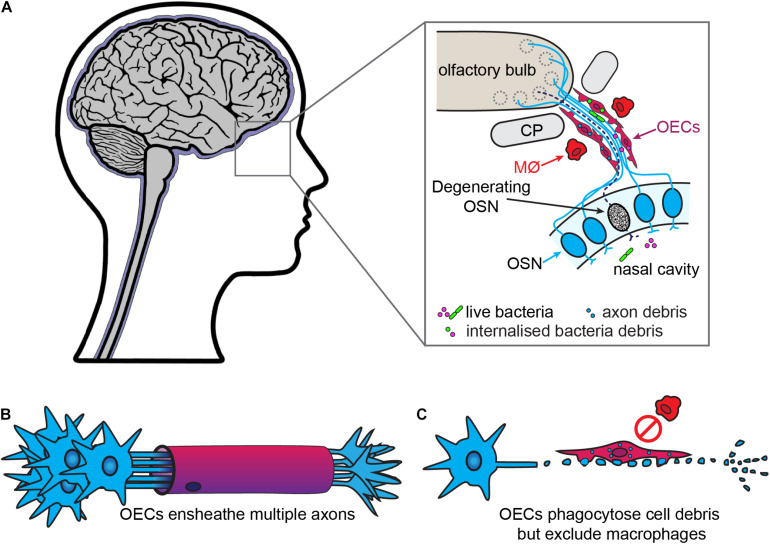
Olfactory ensheathing cells are the primary phagocytes in the olfactory nerve. **(A)** In the olfactory system, olfactory sensory neurons (OSN, blue) project dendrites into the nasal cavity and extend axons into the olfactory bulb. The bundles of olfactory axons are surrounded by OECs. OSNs are constantly turned over and replaced, with the debris from the degenerating axons (dashed line) phagocytosed by OECs. The OECs also provide a line of defense against bacteria from the nasal cavity which penetrate the olfactory nerve, with OECs engulfing the bacteria. Macrophages (MØ) are largely absent from the olfactory nerve. **(B)** In contrast to SCs, OECs ensheathe multiple axons and do not myelinate olfactory axons. **(C)** After injury, OECs phagocytose cell debris but do not recruit macrophages.

Olfactory ensheathing cells are the glia of the primary olfactory nervous system, which consists of the olfactory neuroepithelium, olfactory nerve and outer layer (nerve fiber layer, NFL) of the olfactory bulb (Figure 2). OECs are found throughout this system, except within the superficial neuroepithelium. In addition to the main olfactory system, an accessory olfactory system is present in many animals and is located in the dorsal-caudal region of the olfactory bulb. The primary part of the accessory olfactory nervous system is also populated by OECs (reviewed by Mucignat-Caretta et al., 2012). OECs are somewhat similar to non-myelinating SCs in that they ensheathe bundles of non-myelinated axons (Figure 2). However, olfactory nerve bundles are typically larger than the bundles of unmyelinated peripheral axons ensheathed by SCs (Doucette, 1990; Woodhoo and Sommer, 2008).

In early development, OECs migrate from the neural crest to populate the olfactory placode as early as embryonic day (E9) (in mice) (Katoh et al., 2011; Suzuki and Osumi, 2015), from where they continue to migrate towards the periphery and become part of the olfactory nerve. The olfactory neuroepithelium contains the cell bodies of immature and mature olfactory sensory neurons, basal cells and supporting or sustentacular cells. After differentiation, olfactory sensory neurons start to extend their axons (around E11) into the lamina propria that underlies the olfactory neuroepithelium, where they encounter OECs. As the axons grow towards the emerging olfactory bulb, they are continuously in close contact with OECs (Valverde et al., 1992). Unlike SCs, which accompany developing neurons, OECs migrate ahead of emerging axons (Doucette, 1990; Chuah and Zheng, 1992; Chehrehasa et al., 2010; Ekberg et al., 2012). OECs constitute a structural, tunnel-like support structure for the axons and secrete growth and guidance factors, such as nerve growth factor (NGF), brain derived nerve growth factor (BDNF) and neuregulin (Kafitz and Greer, 1999; Boruch et al., 2001; Woodhall et al., 2001; Lipson et al., 2003; Chung et al., 2004; Feng et al., 2012). Around E12-E13, the developing olfactory nerve bundles fuse with the developing telencephalon and this region then forms the presumptive olfactory bulb (Doucette, 1989) with OECs being limited to the NFL (Valverde et al., 1992). The NFL consists of an outer and an inner layer. OECs located in the outer NFL contribute to the defasciculation of the axons that project into the outer layer of the olfactory bulb, while OECs of the inner NFL mediate sorting and re-fasciculation of these axons into bundles that are projected into specific glomeruli depending on their odorant receptor profile (Mombaerts et al., 1996; Treloar et al., 2002).

These multiple OEC-mediated roles are also important for maintaining normal olfaction both postnatally and during adulthood, as the primary olfactory nervous system is unique in that it regenerates itself during life. When reaching the end of their lifespan, the primary olfactory neurons die, whilst new ones arise from epithelial progenitor cells. In adult animals, olfactory neurons have a lifespan of 1-3 months, with 1-3% of the neurons undergoing apoptosis each day (Graziadei and Graziadei, 1979; Mackaysim and Kittel, 1991). Thus, new olfactory axons are always extending towards the olfactory bulb, with OECs constantly supporting, guiding and sorting these axons (Chuah and Zheng, 1992; Chehrehasa et al., 2010).

Like SCs, OECs are heterogeneous glia, with individual subpopulations exhibiting different properties depending on their anatomical location (reviewed by Ekberg et al., 2012; Ekberg and St John, 2014; Yao et al., 2018). The subtypes of OECs include (i) OECs present in the lamina propria/olfactory nerve, (ii) OECs within the NFL of the olfactory bulb which in turn consists of inner- and outer NFL population and can be even further divided into distinct subpopulations (Windus et al., 2010), and (iii) accessory olfactory bulb OECs. Individual subpopulations of OECs also display differential expression of various molecules *in vivo* (Ekberg et al., 2012; Ekberg and St John, 2014; Perera et al., 2020) as well as differential behaviors regarding cell-cell/cell-axon interactions and phagocytic activity (Windus et al., 2010; Nazareth et al., 2015b).

### OEC Phagocytosis in Normal Physiological Conditions

As in other regions of the nervous system, excess axons and axonal branches that arise during development of the primary olfactory system need to be removed or pruned. In mice, phagocytosis of axon debris by OECs is observed *in vivo* as early as E14.5, and cultured OECs derived from these mice phagocytose olfactory axon debris *in vitro* (Nazareth et al., 2015a). The continuous turnover of the olfactory sensory neuron population in adults means that axons of apoptotic neurons are constantly present in the olfactory nerve, leading to large amounts of axon-derived debris. This debris must be removed so newly born neurons can extend their axons into the olfactory nerve and bulb. OECs are the main phagocytes that remove axon debris arising from apoptotic neurons, whilst only very few macrophages are present in olfactory axon bundles (Figure 2; Su et al., 2013; Nazareth et al., 2015a). In addition to debris, OECs can phagocytose bacteria and are likely important for protecting the olfactory nerve against infection by microbes, as part of their normal physiological function as well as in more severe infections (covered in the next section) (Harris et al., 2009; Herbert et al., 2012; Panni et al., 2013; Dando et al., 2014).

Olfactory neurons undergo cell death via apoptosis upon reaching the end of their lifespan, displaying the “eat me” signal phosphatidylserine. *In vitro* studies have shown that OECs recognize phosphatidylserine on apoptotic neurons via phosphatidylserine receptors prior to engulfment (He et al., 2014; Hao et al., 2017). As discussed earlier, phagocytes usually possess a number of receptors that recognize phosphatidylserine on an apoptotic target; while some receptors directly recognize phosphatidylserine, others require bridging molecules to aid attachment. One such bridging molecule, milk fat globule-EGF factor 8 (MFGE-8), that works with integrin receptors (Hanayama et al., 2002), is expressed by OECs *in vitro* when apoptotic debris is added to the culture (Li et al., 2017). Normal physiological apoptosis is a “silent” process, in which phagocytic cells engulf apoptotic bodies and secrete anti-inflammatory cytokines (such as IL-10 and TGF-β) (Henson, 2017). Indeed, when phagocytosing apoptotic debris derived from neurons, OECs secrete TGF-β1 which may promote phagocytosis via integrin receptors (Li et al., 2017).

Olfactory ensheathing cells isolated from distinct anatomical locations exhibit different phagocytic capacities both *in vivo* and *in vitro* (Nazareth et al., 2015b). One study has shown that acutely isolated OECs from the main olfactory bulb contain more cytoplasmic axon-derived debris than OECs from the accessory bulb. However, after *in vitro* culture, the opposite occurred (Nazareth et al., 2015b). Perhaps the phagocytic activity of accessory OECs, which are less phagocytic than main OECs *in vivo* (possibly due to unknown differences in the requirement for phagocytosis in the main *versus* accessory olfactory nervous system), is more dynamic and susceptible to up-regulation than that of main OECs.

In summary, OECs are efficient and active phagocytes throughout life, including in adulthood. This function differs from that of SCs populating adult peripheral nerves that do not regenerate unless injured. OECs express some key phagocytosis receptors, but may exhibit many more not described to date. Further, different subpopulations of OECs may have different phagocytic abilities. This needs to be further explored especially when determining the best population of OECs to be utilized for transplantation therapies to treat CNS pathologies. Importantly, studies on OEC autophagy are also lacking.

### OEC Phagocytosis – Pathological Conditions

#### Olfactory Nerve Injury

The primary olfactory nervous system is capable of regeneration and repairing itself after most injuries. In animal injury models, both after destruction of the olfactory epithelium (via zinc irrigation or exposure to the drug methimazole) as well as ablation of an olfactory bulb, OECs phagocytose large amounts of debris from degenerated axons (Chuah et al., 1995; Su et al., 2013; Nazareth et al., 2015a). Even in the presence of a small number of invading macrophages after large-scale injury, it is the OECs that contain the vast majority of the internalized debris even after injury (Su et al., 2013; Nazareth et al., 2015a).

Similarly, in the injured *Drosophila* primary olfactory nervous system, ensheathing glia (*Drosophila* glia equivalent to OECs), phagocytose debris. This occurs via *draper/shark/ced-6* pathway (Doherty et al., 2009). The mammalian orthologues MEGF10/engulfment adaptor PTB Domain containing-1 (GULP-1), have been identified to mediate phagocytosis of apoptotic debris by astrocytes after injury or insult such as ischemia (Hamon et al., 2006; Iram et al., 2016; Morizawa et al., 2017). Whether OECs utilize the MEGF-10/GULP-1 pathway to phagocytose apoptotic debris following injury in mammals is yet to be determined.

*In vitro* phagocytic assays have also shown that OECs are capable of taking up debris derived from several types of axons (generated by destruction of axons, thus resembling an injury containing both apoptotic and necrotic bodies) as well as myelin debris (He et al., 2014; Nazareth et al., 2015a, 2020; Khankan et al., 2016; Hao et al., 2017). OECs can, like SCs, actively phagocytose and degrade necrotic cells *in vitro* (also via phosphatidylserine recognition). However, unlike SCs that secrete inflammatory cytokines such as TNF-α and IL-6 post-phagocytosis of these targets, OEC phagocytosis of necrotic bodies does not lead to production of TNF-α, and to only very low levels of IL-6 (Nazareth et al., 2020).

While OECs can effectively engulf and degrade many types of debris *in vitro*, the mechanisms and receptors involved remain mostly unknown, and the phagocytic activities need to be investigated *in vivo.* One study has demonstrated that OECs transplanted into the X-irradiated spinal cord are phagocytic. Despite having a different developmental origin than microglia, these OECs were reported to be “microglia-like,” expressing OX42 (CD11), a microglial marker (Lankford et al., 2008). However, *in vitro* immunolabelling of OECs showed that they do not express this marker under normal physiological conditions, at least not in culture (Hao et al., 2017). In another study, OECs and fibroblasts were transplanted separately into transected mouse spinal cords. Clearance of myelin was significantly more pronounced when OECs were transplanted than after transplantation of fibroblasts (Khankan et al., 2016).

#### Olfactory Nerves and Infection

The olfactory nerve, like the intranasal branches of the trigeminal nerve, is a direct conduit from the external environment to the CNS, providing a potential entry point for pathogens. Some bacteria, including *S. pneumoniae*, *N. meningitidis*, *B. pseudomallei*, and *C. muridarum* (van Ginkel et al., 2003; Sjolinder and Jonsson, 2010; St John et al., 2014; Nazareth et al., 2021) and likely also *C. pneumoniae* (Little et al., 2004, 2014; Boelen et al., 2007), viruses (HSV-1, SARS-CoV2) (Shivkumar et al., 2013; Brann et al., 2020; Meinhardt et al., 2020), and protozoa (*N. fowleri*) (Jarolim et al., 2000; Moseman, 2020) can enter the CNS via the olfactory nerve. Similar to those that can infect CNS via the trigeminal nerve, most of these infectious agents reached the CNS rapidly (within 24-48 h). With the exception of these microorganisms, OECs are considered to be efficient in defending the CNS against microbes that manage to penetrate the olfactory epithelium (Dando et al., 2014). However, epithelial injury may increase susceptibility to invasion, even with microorganisms that do not typically invade the CNS via this path (Harris et al., 2009; Herbert et al., 2012; Walkden et al., 2020).

Olfactory ensheathing cells cultured *in vitro* produce an immune response to bacterial lipopolysaccharide (LPS) and various pathogen associated molecular patterns (PAMPs). OECs also contain TLR-2, TLR-4 and mannose receptors that may aid in recognizing and responding to various pathogenic components (Vincent et al., 2007; Carvalho et al., 2013). OECs challenged with *E. coli in vitro* can endocytose the bacteria, resulting in an inflammatory response, with NF-κB translocation, cytokine growth-regulated oncogene (Gro) and iNOS production (Harris et al., 2009; Panni et al., 2013). OECs display a chemoattraction to heat-killed *E. coli*, which they recognize via TLR-4, and are capable of degrading them by phagocytosis (Leung et al., 2008). However, it has not been documented that OECs actually degrade live *E. coli.* OECs also respond to *Staphylococcus aureus* infection both *in vivo* and *in vitro* with an inflammatory response including secretion of IL-6, TNF-α, NF-κB, and iNOS (Harris et al., 2009; Herbert et al., 2012).

*In vitro* studies have shown that OECs can respond to those bacteria that can invade the olfactory nerve, but that the response is not enough to eliminate the bacteria. Upon challenge with *B. pseudomallei*, cultured OECs can rapidly kill ∼90% of the bacteria, as well as produce a range of cytokines/chemokines (Dando et al., 2016). *B. pseudomallei* can, however, also survive inside some OECs, and like *B. pseudomallei* infection of trigeminal SCs, this can lead to the formation of multinucleated cells (Walkden et al., 2020). *S. pneumoniae* bacteria can be recognized and internalized by OECs after binding to mannose receptors on the cells (Macedo-Ramos et al., 2011; Carvalho et al., 2013), but cannot be degraded by the cells (Macedo-Ramos et al., 2016). One study showed that *S. pneumoniae* suppresses the OEC-mediated immune response by downregulating iNOS production and secretion of growth factors such as NT-3, BDNF and GDNF, which can affect general glial health and function, and thus immune functions (Ruiz-Mendoza et al., 2016). The same study also indicated that *S. pneumoniae-*infected OECs can secrete factors that contribute to microglial apoptosis (Ruiz-Mendoza et al., 2016). Whilst not yet studied *in vivo*, it is possible that that repression of microglial responses could aid infection of the olfactory bulb (and subsequently the rest of the CNS). A recent study showed that OECs can respond to and restrict, but not eliminate, intracellular growth of *C. muridarum*. In fact, OECs responded to these bacteria in a similar manner to macrophages with secretion of a plethora of cyto- and chemokines; the immune response was overall stronger than for trigeminal SCs (Nazareth et al., 2021). *Chlamydiae* bacteria have previously been widely reported to survive and persist for very long periods in professional phagocytes, in particular in macrophages (reviewed by Chen et al., 2019; Wong et al., 2019). Whether *C. muridarum* can persist in OECs in the long-term and if this this may contribute to infection of the CNS remains to be investigated.

In summary, OECs are the main phagocytes in the olfactory system and play an active role in phagocytic clearance of axon debris throughout life, as well as after an injury. Also, being situated near the external environment in the nasal cavity, OECs are equipped to protect against microbial challenges. Certain infectious agents can manipulate the phagocytic pathway of the cells and survive intracellularly, similarly to those that can resist killing by SCs (Macedo-Ramos et al., 2011, 2016; Mutso et al., 2020). However, much is lacking regarding our knowledge of the molecular mechanisms involved in OEC-mediated phagocytosis; studies in transgenic mice lacking key phagocytic receptors are particularly warranted. Identifying and understanding the differences between physiological phagocytosis and infection-driven phagocytosis will help identify ways to protect the brain from pathogens that may invade the CNS via the olfactory nerve, as previously discussed for trigeminal nerve infection.

## Discussion

### Key Differences Between SC- and OEC- Mediated Phagocytosis

Over the years there has been a debate about which peripheral glia, SCs or OECs, are more suitable to treat CNS pathologies. An ideal candidate to treat nervous system injury would need to be an efficient phagocyte, capable of clearing cellular and myelin debris without production of adverse inflammatory response. In addition, it would also require modulating the existent inflammatory environment and continue secreting nerve growth factors to help regeneration. Hence it is important to discuss how SCs and OECs may differ in these aspects as it may enable to decide on which cell type may possess the best therapeutic potential.

In the past, OECs and SCs were thought to be relatively similar (Wewetzer et al., 2002; Ulrich et al., 2014); OECs were even called “olfactory SCs.” However, studies over the last two decades have revealed that the two cell types are unique and have clearly distinct genetic profiles (Franssen et al., 2008; Perera et al., 2020). Gene ontology studies comparing transcriptomes of the two cell types showed that cultured OECs display a higher level of expression of genes related to tissue repair and regeneration in particular those required for phagocytosis and degradation of targets (Vincent et al., 2005; Franssen et al., 2008). Similarly, OECs have been shown to be more efficient phagocytes than SCs of necrotic/myelin debris *in vitro* (Nazareth et al., 2020). When challenged with various infectious agents and pathogen-derived ligands *in vitro* OECs have been found less prone to infection, and produce a stronger immune response compared to SCs (Vincent et al., 2007; Walkden et al., 2020; Nazareth et al., 2021).

Schwann cell phagocytosis in peripheral nerves appears to occur on a significant scale only in pathological conditions. While SCs participate in the initial debris removal of the injured nerve, other cells like perineurial glia and resident macrophages may also be involved in this process (Mueller et al., 2003; Lewis and Kucenas, 2014; Wang et al., 2020). Further they produce a range of pro-inflammatory mediators that recruit immune cells, particularly macrophages, to aid in debris clearing (Dubovy et al., 2014; Hartlehnert et al., 2017). Unlike SCs, OECs are a part of a constantly regenerating nerve, requiring rapid and efficient phagocytosis of axons from apoptotic neurons to prevent inflammation. OECs are the key cells performing this function without recruiting macrophages (Chehrehasa et al., 2014; Nazareth et al., 2015a; Murtaza et al., 2019). Whilst the mechanisms behind this key difference between OECs and SCs is mostly unknown, macrophage migration inhibitory factor (MIF), which is secreted by both OECs and macrophages, mediates segregation between the two cell types at least *in vitro* (Wright et al., 2020).

Inflammation is in general considered to hamper neural regeneration. OECs can produce a range of pro-inflammatory cytokines and chemokines both after injury and infection, but simultaneously promote rather than inhibit nervous system regeneration (Pastrana et al., 2006; Vincent et al., 2007; Franssen et al., 2008; Roet et al., 2011). One reason for this may be that OECs challenged with various inflammatory stimuli continue proliferating while promoting neurite outgrowth, with an increased phagocytic activity (Lankford et al., 2008; He et al., 2014; Roet and Verhaagen, 2014; Hao et al., 2017). Another reason may be that SCs can lose their plasticity when present in a chronically inflamed environment (Joshi et al., 2016) and this may affect their ability to perform phagocytosis and aid regeneration post-transplantation. However, the specific milieu at a CNS injury site, especially in the chronic stages, is a very complex inflamed environment, with reactive astrocytes, invading immune cells, growth-inhibitory factors, necrotic cells and large amounts of debris. It is unknown if both OECs and SCs continue to be efficient phagocytes in this complex environment, whilst also promoting regeneration. While phagocytosis of apoptotic targets is a silent event, phagocytosis of necrotic and myelin debris is usually followed by production of pro-inflammatory cytokines (Nazareth et al., 2020). For therapeutic purposes, however, it is critical that a strong pro-inflammatory response does not occur (in particular after transplantation into CNS injury sites, which are already hostile and pro-inflammatory). OECs been suggested to be immunomodulatory and/or in fact decrease inflammation in the host tissue (Lopez-Vales et al., 2004; Huo et al., 2012; Khankan et al., 2016; Xie et al., 2019; Zhang et al., 2021). One study showed that whilst SCs may have had some beneficial effects on the number of invading macrophages, they were unable to modulate the overall inflammatory environment at the CNS injury site (Pearse et al., 2018), but more studies on SCs are required. Thus, we need to increase our understanding of the mechanisms involved in both OEC and SC-mediated phagocytosis.

As OECs and SCs may have different but still complementary phagocytic abilities, co-transplantation of both cells may be an interesting option. Few studies have to date investigated co-transplantation of OECs and SCs to treat nervous system injuries (Ramón-Cueto et al., 1998; Takami et al., 2002; Pearse et al., 2004; Fouad et al., 2005; Au et al., 2007; You et al., 2011; Sun et al., 2013; Chen et al., 2014; Zhang et al., 2017), however the outcomes have been variable. One study looking at transplantation of either SCs, OECs or combination treatment into rat thoracic spinal cord after a moderate contusion injury reported that all three treatment groups presented a decrease in cavitation at the injury site and increase in number of myelinated axons (Takami et al., 2002). However, the number of myelinated axons were higher in SC or combination group than OECs alone. There was also a significant recovery in locomotor function in the SC alone treatment than the other two groups, thus indicating that the SC alone treatment produced best outcomes post-transplantation (Takami et al., 2002). However, interestingly there was an increase in accumulation of reactive astrocytes in the SC containing groups (Takami et al., 2002). In contrast, two other groups reported no difference between SCs only, OEC only and combination treatment (Pearse et al., 2004; Chen et al., 2014). A clinical study in patients with chronic complete SCI reported functional recovery in all groups receiving cell treatments (SCs, OECs, or co-transplantation) compared to no treatment group (Chen et al., 2014). However, no differences in recovery were observed between the three groups receiving glia transplantation. Similarly, in a rat model of thoracic contusive injury, while nerve growth was observed after transplantation of the two peripheral glia and in the co-transplantation group, no differences were reported amongst the three groups (Pearse et al., 2004). However, the authors also reported that locomotor recovery was greatly enhanced when methylprednisolone (a corticosteroid) and IL-10 were administered prior transplantation of glia (Pearse et al., 2004). Similar outcomes were also reported in complete spinal transection studies in adult rats. While cell grafts consisting of OEC and SC bridges improved forelimb and hindlimb movement along with an increase in number of myelinated axons and serotonergic fibers than non-treated controls, supplementing cell grafts with chondroitinase improved overall outcomes (Fouad et al., 2005). On the other hand, some studies have reported improved outcomes in nervous system injury models with combination treatment with SCs and OECs than either cell alone. One PNS study showed that co-transplantation promoted both axonal regeneration and functional repair after sciatic nerve injury in rats compared to transplantation of either cell type alone (You et al., 2011). Similarly, a CNS study showed that co-transplantation of OEC and SCs into the contused rat spinal cord resulted in better modulation of the inflammatory response (lower numbers of reactive astrocytes, reduced infiltration by immune cells and shift in microglia/macrophages to more anti-inflammatory phenotypes), along with improved motor function (Zhang et al., 2017). The authors also report greater distribution of glia at the site of injury when combined. This is not surprising as previous studies have reported than introducing OECs into an injured spinal cord increased migration of SCs along with improved axonal regeneration (Ramón-Cueto et al., 1998; Au et al., 2007). However, none of these co-transplantation studies have investigated the phagocytic clearance of cellular and myelin debris in this combination therapy. As we know that rapid clearance of debris is crucial for repair following nervous system injuries, an understanding of this crucial function will help us achieve the best outcomes from a potential co-transplantation therapy.

### Stimulating Phagocytosis of Peripheral Glia to Treat Neural Injuries

Stimulating phagocytosis of endogenous or transplanted peripheral glia in nerve injuries may aid the overall process of regeneration and recovery (Wright et al., 2018). Several animal studies have shown that stimulating SC clearance of myelin debris can improve outcomes after injury. For example, supplying NGF after sciatic nerve crush injury in mice enhanced SC uptake and degradation of myelin both via autophagy and phagocytosis, leading to improved nerve regeneration (Li et al., 2020). Application of pharmacological agents such as rapamycin (a lipophilic macrolide drug which promotes SC autophagy) (Huang et al., 2016), antineoplastic drug Epothilone B (Zhou et al., 2020), as well natural compounds such as curcumin (Liu et al., 2016; Zhao et al., 2017), and resveratrol (Zhang J.Y. et al., 2020) have altered SC autophagic flux to enhance clearance of myelin and improve myelination. Specific enhancement of SC autophagy has also been explored to treat various peripheral neuropathies. Administration of rapamycin in mice during early stages of Wallerian degeneration increased SC autophagic flux which promoted myelin compaction, which occurred along with reduced inflammatory responses. This aided long-lasting analgesic effects and prevented development of chronic pain (Marinelli et al., 2014). Peripheral neuropathies are also characterized by either abnormal protein expression or mis-folding of proteins which aggregate within SCs. One such protein is peripheral myelin promoter 22 (PMP 22) that has been reported to be mis-expressed and aggregated within SCs especially in type 1 CMT (van Paassen et al., 2019). Treatment of SCs with rapamycin (Rangaraju and Notterpek, 2011), suberoylanilide hydroxamic acid (SAHA), 17-allylamino-17-demethoxy-geldanamycin (17-AAG) or clonazepam (Watanabe et al., 2010) facilitated autophagy, resulting in breakdown and disposal of these aggregated unfolded proteins *in vitro*. However, *in vivo* studies in a mouse model of CMT1A linked with PMP22 aggregation showed that although rapamycin promoted peripheral nerve myelination it did not improve the overall neuromuscular function in these animals (Nicks et al., 2014). The field would, however, strongly benefit from more studies directed towards stimulating SC phagocytosis rather than just autophagy for therapeutic purposes.

Several studies have to date identified compounds that can stimulate OEC phagocytosis (as well as autophagy – a topic which remains to be studied in itself). We, along with laboratories around the world, have identified various agents that can increase OEC phagocytosis, including the natural products 2-methoxy-1,4-naphthoquinone (Chen et al., 2020), the serrulatane diterpenoids 3-acetoxy-7,8-dihydroxyserrulat-14-en-19-oic acid and 3,7,8-trihydroxyserrulat-14-en-19-oic acid (Chen et al., 2018), curcumin (Velasquez et al., 2014); curcumin in combination with *E. coli*-derived LPS (Hao et al., 2017), and anti-inflammatory cytokine TGF-β (Li et al., 2017). *In vitro* injury assays (neurons co-cultured with OECs accompanied by neuronal debris addition), showed that increased OEC clearance of debris due to application of stimuli (LPS + curcumin Hao et al., 2017 or TGF-βLi et al., 2017) promoted neuronal survival. A recent study in a rat spinal cord injury model reported that transplanted OECs that had been pre-treated with curcumin led to better functional recovery and axonal regrowth than transplantation with control OECs (Guo et al., 2020). The stimulated cells secreted larger amounts of growth factors *in vivo* along with improved immunomodulation of the injury site. The same study also reported that the OECs stimulated with curcumin exhibited increased phosphatidylserine receptor expression (Guo et al., 2020), suggesting that increased phagocytic activity may have aided the overall therapeutic outcomes. Thus, stimulating glial phagocytosis may improve neural repair therapies.

### Stimulating Peripheral Glia to Clear Pathogens

Peripheral nerves can allow microbes to enter the CNS, bypassing the BBB and the blood- cerebrospinal fluid barrier. The ability to infect and survive within glia are suggested to be a key strategy by which certain infectious agents can gain access to these nerves (Dando et al., 2014), but further mechanistic and *in vivo* studies are needed, which can reveal drug targets. In particular, the phagocytic phase in which glia degrade microorganisms needs attention. We also need to understand specific risk factors of CNS infections via cranial nerves, such as epithelial injury and long-term use of antibiotics, which may promote persistent infection. Another important research topic is to determine how infections can re-activate after being latent or dormant in the nervous system. It is well known that bacteria can persist within phagocytic cells to later escape and cause infection under favorable conditions (Bastidas et al., 2013), but very little is known regarding this phenomenon in glia. We also need to understand the long-term consequences of nervous system infection, in which glia have important roles. These may include neurodegeneration; for example, the connection between late-onset dementia and (1) infectious agents (including both *C. pneumoniae* and HSV-1) and (2) genetic factors such as the apolipoprotein ε4 (*ApoEε4*) allele, which is associated with impaired glial phagocytosis, is becoming increasingly evident (Itzhaki et al., 2016; Balin et al., 2018; Woods et al., 2020).

## Concluding Remarks

Glia of the peripheral nervous system are strongly phagocytic cells during development, in the adult and after injury or infection. Yet, depending on their location the glia have different functions and engulf both “self” and “non-self” targets. In the developing and adult CNS, glia play an important role in the phagocytic clearance of various targets. However, in the PNS under physiological conditions not much is known about the role of SC phagocytosis in the maintenance of homoeostasis, particularly in mammals. In the healthy adult, damage to most peripheral nerves is limited and the need for phagocytosis by SCs is low. In contrast, the olfactory nerve is constantly being replenished and is also subjected to microbe invasion; thus, OECs are required to constantly phagocytose cell debris and be ready to respond to infectious agents. After injury, SCs clear cellular and myelin debris, however they enlist the help of other cells including perineurial glia and recruit macrophages to aid clearance and repair. On the other hand, OECs are largely responsible for phagocytosis and do not appear to recruit macrophages. While both SCs and OECs have a capacity for phagocytosis, the mechanisms that govern this process are largely unknown. Identifying the molecular and cellular mechanisms that drive phagocytosis of various targets by peripheral glial will then allow us to exploit this function. Stimulating SCs and OECs to clear cellular and myelin debris will help improve repair after injury or will aid better infection prevention/treatments. Understanding the differences in the phagocytic characteristics between SCs and OECs is also important for development of transplantation therapies to repair neural injuries as it may be possible to exploit key benefits of each cell type. Overall, increasing our understanding of glial phagocytosis may lead to the design of therapies to treat injuries and diseases of the nervous system.

## Author Contributions

LN performed the literature search and drafted the manuscript. JE provided the overall supervision. All authors reviewed and edited the manuscript.

## Conflict of Interest

The authors declare that the research was conducted in the absence of any commercial or financial relationships that could be construed as a potential conflict of interest.
